# Physical Activity and Lifestyle Behaviors in Children and Adolescents

**DOI:** 10.3390/children11111403

**Published:** 2024-11-20

**Authors:** Alexandre Aparecido de Almeida, Matias Noll

**Affiliations:** 1 Instituto Federal de Educação, Ciência e Tecnologia do Tocantins, Campus Araguatins, Araguatins 77950-000, Brazil; 2 Instituto Federal Goiano, Campus Ceres, Ceres 76300-000, Brazil; matias.noll@ifgoiano.edu.br; 3 Faculdade de Nutrição, Universidade Federal de Goiás, Goiânia 74605-080, Brazil

A sedentary lifestyle, unfavorable body composition, and low muscle strength are strong predictors of morbidity and mortality and an independent determinant contributing to the development of many chronic diseases [1,2]. In contrast, adopting healthy lifestyle habits, including physical activity, provides people with several physical, cognitive, and mental benefits. In this regard, meta-analytics studies support the beneficial effects of physical activity interventions on cognitive functioning, particularly in the domains of attention, inhibition, working memory, and cognitive performance during early childhood (3–6 years) [3]; academic achievement [4]; and positive changes in the body mass index and percent fat [5] in youth. Despite these well-known benefits of an active lifestyle, a considerable proportion of children and adolescents do not reach the daily physical activity recommendations [6,7]. Furthermore, whenever such a young person engages in harmful lifestyle habits, the risks of developing health problems increase.

In 2010, more than a decade ago, the World Health Organization (WHO) pointed out that children and adolescents engage in a minimum of 60 minutes of moderate-to-vigorous physical activity (MVPA) daily, with durations exceeding 60 minutes per day providing additional positive health impacts [8]. These activities can be diverse, including transportation, recreation, or planned exercise into family, school, or community context [8]. Even so, the majority of adolescents fail to meet recommended physical activity guidelines, thereby jeopardizing both their current and future health. A recent global study shows that 81% of adolescents spend less than one hour a day in moderate- to vigorous-intensity physical activity, with girls (85%) being more inactive than boys (approximately 78%) globaly [6].

Another important aspect of life is the adoption of healthy lifestyle habits that include physical activity practices, but they are not restricted to it alone. A healthy lifestyle, i.e., a way of living that lowers the risk of being seriously ill or dying early [9] that profoundly influences health includes maintaining a healthy body weight, Abstaining from cigarette smoking, as well as adhering to balanced nutritional habits and other health-promoting practices [10]. As childhood and adolescence are important periods of youth development, it is important to adopt healthy behaviors by providing opportunities to increase and enhance positive lifestyle behaviors. Like this, studies have pointed to the insertion of playgrounds in schools [11], wearable activity tracker use [12], and school-based physical activity programs [13] as approaches to enhance physical activity levels. In addition, school-based interventions for promoting food and nutrition literacy have been useful in improving food and nutritional knowledge [14].

This editorial refers to the closing of the Special Issue “Physical Activity and Lifestyle Behaviors in Children and Adolescents” in the journal *Children*. Therefore, we aim to highlight the current knowledge in research relevant to impacts of involvement in physical activity practices and lifestyle habits in young people, highlighting the impacts of lifestyle habits, good or harmful, on different aspects of life. Papers in this issue cover most of these themes and offer recent viewpoints about the questions that are of interest to the field. In total, ten papers were accepted for publication and included in this Special Issue (all were original articles).

**A brief background of published articles**

Studies published in this Special Issue covered areas such as the acute effects of physical exercise in youth with special conditions (ADHD); body image and lifestyle behaviors; the effects of moderate-to-vigorous physical activity on general well-being; the effects of physical-literacy-based online education on physical fitness; the effects of a special game (“Clock Motor Game”) on children with learning difficulties; the impact of adverse events and aerobic exercise (step aerobics) on sleep quality; the relationships between mental health indicators and internet use; school-based physical activity strategies to increase physical activity; the effects of growth and body surface area; and the effect of a patient-reported outcome measurement information system on cardiopulmonary fitness.

Studies were conducted in countries such as United States of America (2), China (2), Croatia (1), Canada (1), Lithuania (1), Spain (1), Greece (1), and Tunisia (1). Only one study exclusively considered girls. Nine studies included both girls and boys in their samples. The total sample analyzed included 5844 volunteers (2585 boys, 44.23%; and 3259 girls, 55.77%) with mean ages between 6.70 and 18.40 years (minimum: 6 years, maximum: 19 years).

Below we summarize the studies published in the issue.

The first contribution (contribution 1) described by us is talking about a very little explored subject in relation to the effects of physical exercise on intellectual/behavioral conditions: the impact of acute physical exercise on Attention Deficit/Hyperactivity Disorder (ADHD). In general, studies exploring this issue have focused on adult populations and chronic physical exercise effects. Using a rapid crossing of terms “ADHD acute physical exercise” in the PubMed database, only fifty-two published papers were found. When limited by “Child: 6–12 years and Adolescent: 13–18 years” filters, this number was reduced by half (exactly 26 papers). This low number of studies emphasizes the importance of further research on this topic. The above-mentioned contribution investigated twenty-two children aged 6 to 11 years. Using a randomized design, children were allocated to a walking intervention group (n = 7), a standing group (n = 7), or a sitting condition control group (n = 8). Inhibitory control was assessed using the Stroop Color and Word Test, Children’s Version, and problem solving and cognitive flexibility were assessed using the Wisconsin Card Sorting Task (WCST). Principal results showed that walking had a lower effect on the inhibition scores compared to the standing and sitting groups (non-significant difference). Although non-statistically significant, remaining standing changed all five classes of the WCST compared to the walking and sitting conditions. Thereby, these results should be interpreted with caution; they have the potential to support public strategies in a college setting. However, more definitive recommendations can be obtained from larger-scale studies in children with ADHD.

Positive or negative self-perceptions and attitudes concerning their body may play an significant role in adolescent health, resulting in the adoption of a healthy or unhealthy lifestyle [15,16,17]. In this sense, categorized by sex and body weight status, contribution 2 investigated the relation between body size dissatisfaction (BSD) and body size perception (BSP). This paper also explored the associations between lifestyle behaviors and body image variables in 301 adolescent boys and girls (14.1 ± 1.1 years old). To determine BSP and BSD scores, the figural rating scale [18] was used. Lifestyle behaviors such as ingesting of vegetables/fruits and dairy products, hours per week or weekends spent using a computer and watching television, sleep duration, and self-reported wake-up times on weekends and on school days were obtained using online questionnaires. Among normal and overweight/obese (OW/OB) girls’ subgroups, positive relations were found between BSP and BSD data. Tending to perceive themselves as thinner was observed in adolescents who were OW/OB. Adolescents who underestimate their body size but are satisfied with their body size had a lower BMI, less screen time, and slept more compared to those who underestimate their body size and are dissatisfied with their bodies. Adolescents who are overweight or living with obesity undervalued their body size. Girls were more satisfied with their body size while underevaluating their body size. The fact that they undervalued their own body size but were pleased with it may be a protection factor against unhealthy behaviors in this group of adolescents.

Adolescents often experience many psychological challenges [19]. Using a statistical procedure, structural equation modeling, Chen and colleagues (contribution 3) investigated the relation between moderate-to-vigorous physical activity practice and self-disclosure, social anxiety, and social avoidance in a sample of Chinese adolescents. The results indicated that adolescents engaged in moderate to high-intensity physical activities exhibited lower levels of social anxiety. Adolescents with the highest disposition for self-disclosure demonstrated inferior levels of social avoidance. In brief, regular physical activity practices can reduce social anxiety levels, enhance the willingness to self-disclose and decrease social avoidance behaviors.

Moving to the fourth contribution, an adequate physical activity level (PA) is an important determinant of health conditions and a lifestyle medicine [20,21]. Nevertheless, with school closures during the COVID-19 pandemic, younger schoolchildren and adolescents showed an abrupt and heavy decline in all forms of physical activity [22]. Thus, this contribution evaluates the efficacy of specific physical literacy (PL) intervention on physical fitness (PF) scores of students during the COVID-19 phases. Four hundred and twenty-three Croatian high school adolescents were volunteers in an intervention consisting of a weekly distance-learning program conducted over a period of twelve weeks. The intervention included videos about general PL and nutrition. Measurements such as body mass, height, body mass index (BMI), long jump, sit and reach, sit-ups, and pacemaker tests were performed. The twelve-week educational intervention produced positive changes in BMI and cardiorespiratory fitness. The results pointed out that the program had more positive impact to girls.

The fifth contribution explored the relation of the “Clock Motor Game” and “Reading and Recording of Time” (RRT) in kids with mathematical learning difficulties (MLDs). Two hundred and thirty-two kids from ten Tunisian colleges were included. All participants were identified as having MLDs. Children were evaluated at several times [before the intervention (T0), directly later (Ti1, Ti2, Ti3, Ti4, Ti5, and Ti6), and after five weeks (T2)]. Briefly, a circular clock is drawn on the ground and the children placed outside it. Upon receiving a signal, they enter the 'hallway' of the clock, running in the way specified by the instructor. The instructor announces an oral or visual time, and the kids are required to enter the clock and position themselves along the lines corresponding to the stated time. The test finished at the moment that the group organizes quickly and successfully at the announced time. In a general form, the results showed that the experimental group exhibited greater improvements in the “Reading and Recording of Time” compared to the control group.

Adverse events are recognized as cause of bad sleep quality [23]. In contrast, it has been postulated that exercise ameliorates pathophysiological and psychiatric sleep disorders [24]. Using an online survey, demographic information, the adolescent self-rating life events checklist (ASLEC), the Self-rating Anxiety Scale (SAS), and the Pittsburgh Sleep Quality Index (PSQI), contribution 6 evaluated the impact of adverse events and 12-week group step aerobics on sleep quality in Chinese adolescents using statistical modeling. The exercise protocol consisted of performing step aerobics, 75 minutes per session, four times per week over twelve weeks. Importantly, the PSQI scores of the exercise group were lower starting in the eighth week and remain so until the end of the protocol. In conclusion, adverse events had a nonlinearly impact on the sleep quality of these younger. In addition, twelve weeks of this exercise program had a 92.5% likelihood of improving sleep quality.

Entitled “Lifestyle Habits Related to Internet Use in Adolescents: Relationships between Wellness, Happiness, and Mental Health”, the seventh contribution investigated the relationships between internet use and wellness, happiness, and mental health in adolescents. One thousand four hundred and twelve adolescents aged 12 to 16 (14.41 ± 1.20) years participated in the study. Sociodemographic data, emotional and behavioral problems, and problematic internet use (internet addiction) were measured by self-reported questionnaires. Correlation coefficients and linear regression models were used to investigate associations between measures. An analysis of variance was used to compare the groups. Weak to moderated positive correlations were observed between the Internet Addiction Test and internalizing and externalizing strong point and worries scores. It was found that long internet use is a grave issue among young people. Internet addiction was associated with negative feelings, unhappiness, and poor emotion regulation.

Do you want to improve physical activity level? If your response is “yes”, schools appear to be a good local for reaching all young people students individually [25] and growth the physical activity time [26]. The eighth paper measured the effect of an educational program (PEDAL) involving active desks (bike desks) during school time on the physical activity, physical condition, and academic scores results of Spanish students in the fourth year of secondary education (approximately 15-year-olds). Cardiorespiratory fitness (assessed using a 20 m shuttle run test), physical activity levels (International Physical Activity Questionnaire Short Form), language competence, and attention and concentration (measured using the d2 Test of Attention) were measures. Thirty-five active lessons lasting 55 min were applied in the PEDAL program. This intervention lasted 10 weeks. The PEDAL and control groups impacted cardiorespiratory fitness. No significant differences were found between the groups at the conclusion of the program. Only the PEDAL group showed increases in physical activity levels and energy expenditure at the end of the experiment. Language competence, attention, and concentration improved significantly after intervention in both groups. Briefly, the presented bike desk educational intervention caused no negative academic interference.

The performance of young swimmers is the result of a multifactorial process that is influenced by various factors, as anthropometric and training features, physical growth, and/or biological maturation [27]. Thinking about it, the author of contribution 9 evaluated biological maturation and body surface area effects on the cardiopulmonary fitness indicators of preadolescent female swimmers. Preadolescent female swimmers (N = 30, age 13.4 ± 1.0 years) participated in this study. Body composition was assessed using whole-body bioelectrical impedance analysis. The surface area was calculated. Biological maturation was recorded by a female doctor. Cardiopulmonary exercise testing was performed using an electronic cycle ergometer. Except for the correlation between breath frequency and Tanner stages and the ratio between ventilation and body surface area and Tanner stages that were negatives (r values ranged of −0.693 and −0.387, respectively), all other correlations were positives (r values ranged of 0.541 and 0.961). Therefore, girls with reduced body area and biological maturation stage had smaller values of maximal oxygen uptake and higher respiratory work.

Finally, the last paper of this Special Issue (contribution 10) assesses the convergent validity of the Patient-Reported Outcomes Measurement Information System (PROMIS) Pediatric Physical Activity item bank. PROMIS is a new valid instrument to assess the self-reported bouts of moderate to rigorous physical activity in childhood. The authors explored its validity by comparing the relationship of the PROMIS results with cardiorespiratory fitness and self-efficacy for physical activity (PASES) in one hundred eighty-two children and adolescents between 8 and 18 years of age. Volunteers completed questionnaires about PROMIS, self-efficacy in physical activity, and a pediatric step test to measure cardiorespiratory fitness. The results showed a weak but significant negative correlation of PROMIS Pediatric PA scores with the heart rate one minute after completing the step test (r = −0.23) and the PROMIS Pediatric PA scores with the sum of PASES scores (r = 0.27). Like this, the convergent validity of PROMIS was verified with the step test results; as the observed relationships were weak, this may highlight the distinct domains used to characterize physical activity and physical fitness.

**Future directions and conclusions**

Papers in this Special Issue covered considerable areas of the lives of young people such as the acute effects of physical exercise; body image and lifestyle behaviors; the effects of moderate-to-vigorous physical activity on general well-being; the effects of physical literacy programs on motor performance; the effects of a special game on children with learning difficulties, etc. However, more longitudinal studies are necessary to determine how many healthy life habits can exert a positive influence on the health of young people. Furthermore, studies can investigate the impact that healthy life habits exert on the lives of children and adolescents with special conditions such as autism spectrum disorder, Down syndrome, and cerebral palsy. Another issue that needs further investigation is the effects of high-intensity interval training, in its different forms (e.g., SIT, HIIT), on various aspects of infancy and adolescence. In addition, more attention needs to be paid to research involving the physical activity and lifestyle habits of children and adolescents in situations of family neglect, poverty, and among refugees to provide support for more concrete and effective interventions for people in these situations. Finally, more studies on young people investigating the impacts of physical activity and lifestyle habits on the cognitive, academic and behavioral aspects are needed.
